# Structural and Functional Biomechanical Classifications of the Discoid Meniscus

**DOI:** 10.3390/bioengineering13091063

**Published:** 2026-09-13

**Authors:** Sarah Lu, Maria Tuca, Giulia Beltrame, Ariana I. Matarangas, Austin G. Simpson, Daniel Green

**Affiliations:** 1 Hospital for Special Surgery, New York, NY 10021, USA; lusa@hss.edu (S.L.); beltramegiulia.gb@gmail.com (G.B.); amatarangas21@gmail.com (A.I.M.); 2 Hospital Clinico Mutual de Seguridad, Santiago 9160000, Chile; mtuca@alemana.cl

**Keywords:** discoid meniscus, meniscus classification, PRiSM classification, pediatric knee, meniscal preservation, arthroscopy

## Abstract

The discoid meniscus is a morphologic variant associated with altered biomechanics and an increased risk of tearing, yet the systems used to classify it have historically emphasized shape over function. This review examines how classification of the discoid meniscus has evolved, from early morphologic schemes such as the Watanabe classification to more comprehensive frameworks that account for peripheral rim stability, tear characteristics, and functional relevance. We discuss later systems proposed by Klingele, Good, and Yang, which recognized rim stability and tear patterns as key determinants of symptoms and surgical management, and we describe how the PRiSM classification consolidates these advances into a standardized, reproducible system incorporating morphology, thickness, stability, and tear characteristics. We further frame the discoid meniscus as a biomechanical entity in which disorganized collagen, abnormal peripheral attachment, and impaired hoop-stress transmission shape tissue behavior and healing potential. Finally, we argue that future classification systems should integrate morphologic, biomechanical, compositional, and imaging biomarkers to better characterize tissue quality, predict repair outcomes, and guide patient-specific, tissue-preserving treatment. Uniting structure and function within a single framework may improve prognostication and surgical planning.

## 1. Introduction

The menisci are crescent-shaped fibrocartilaginous structures anchored to the tibial plateau by anterior and posterior roots. Their highly organized collagen architecture converts axial loads into circumferential hoop stresses, which are transmitted to the subchondral bone through the root attachments. Continuous, tightly packed circumferential collagen bundles form the main load-bearing framework, while interdigitating radial tie fibers stabilize these bundles and limit longitudinal splitting. This structural organization is complemented by a gradual and seamless transition from the outer, circumferentially oriented region to the inner, more proteoglycan-rich and cartilage-like region, resulting in a functionally graded material optimized for load transmission [1].

The discoid meniscus (DM) represents a congenital variant, typically involving the lateral meniscus, comprising a spectrum of meniscal shapes and degrees of instability. It has been reported in 1% to 5% of the Caucasian population, and in 10–15% of the Asian population. Internationally, the prevalence of bilateral discoid menisci has been estimated to range between 15% and 25% of all discoid menisci diagnoses [2,3]. Whereas the normal lateral meniscus covers approximately 60–70% of the lateral tibial plateau, a complete discoid lateral meniscus may cover up to 100% [4]. A thickened, disk-shaped meniscus with central hypertrophy was first described in early anatomical literature and is discussed in historical reviews of the discoid meniscus [5,6]. Nevertheless the DM should not be regarded merely as a morphological variant. Collagen ultrastructure varies considerably among discoid lateral menisci, with some specimens retaining a nearly normal architecture. Greater collagen disorganization and altered fibril density appear to be associated with the presence and complexity of meniscal tears [7]. In the Wrisberg variant, the absence of the posterior tibial attachment causes hypermobility and may result in complete loss of effective hoop-stress transmission [6]. In other variants, abnormal peripheral attachments and thickening of the popliteal hiatus region may also contribute to instability [4]. These structural and attachment abnormalities may reduce tissue resilience and alter meniscal displacement and load transmission, thereby increasing susceptibility to tearing [7]. Clinical presentation of a discoid meniscus may be asymptomatic or symptomatic, with variability based on patient age and activity level. When symptomatic, approximately one third of patients reported mechanical symptoms such as locking and snapping [8]. Symptoms may arise following an acute traumatic event or develop insidiously over time. On physical examination, findings may include joint effusion and joint line tenderness. Mechanical symptoms can often be reproduced with meniscal provocative maneuvers, such as the McMurray, Apley grind, and Thessaly tests [9].

**Figure 1:**
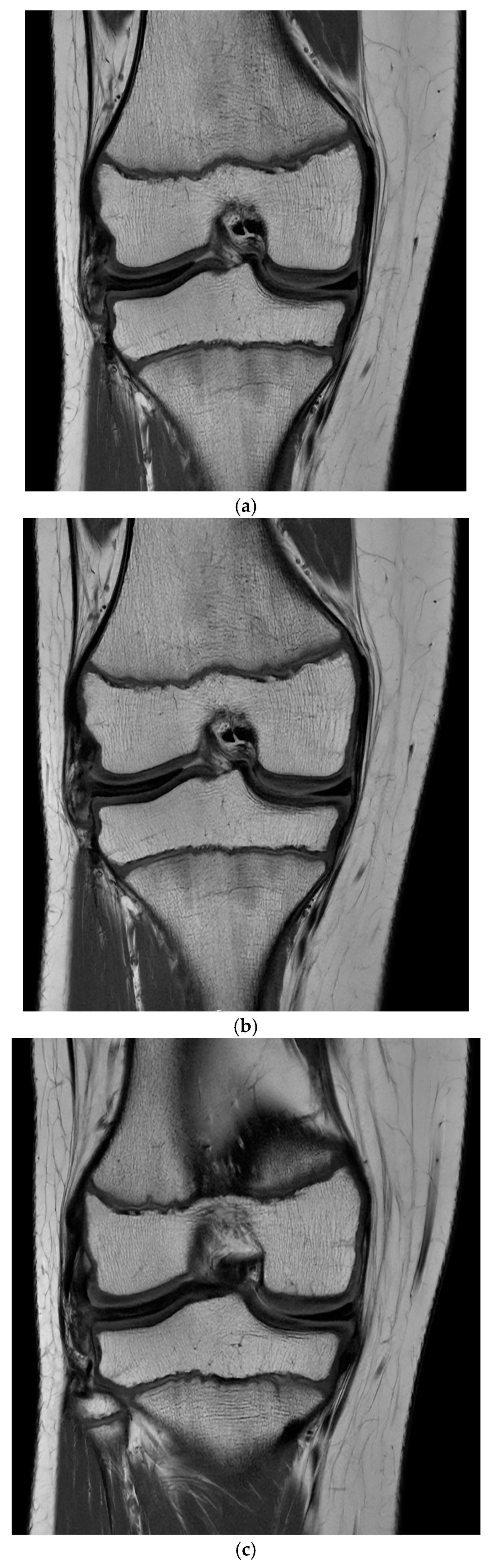
Three consecutive coronal MR images demonstrating the “Bow-tie” sign in the discoid meniscus and a horizontal tear in the meniscus body, with (**a**) being the most anterior, (**b**) an intermediate slice showing continuity of the meniscal body, and (**c**) being the most posterior slice.

Magnetic resonance imaging (MRI) plays a key role in the diagnosis of discoid meniscus, with previous studies reporting a sensitivity of approximately 87% and specificity of 84% for identifying discoid meniscus [10]. MRI is indicated in patients with suspected symptomatic meniscal pathology. A classic radiographic feature is the “bow-tie sign,” defined by continuous visualization of the meniscus on three or more consecutive sagittal images (Figure 1). Additional MRI findings include a thickened, flattened meniscus on sagittal views and meniscal tissue extending across the lateral compartment on coronal images, reflecting the abnormal morphology of the discoid meniscus.

Although previous reviews have comprehensively addressed the morphology, diagnosis, and management of the discoid meniscus, classification has recently evolved beyond traditional morphology-based systems. Newer frameworks incorporate peripheral stability, tear characteristics, meniscal dimensions, and imaging findings, reflecting variables that may directly influence tissue preservation, reparability, and mechanical competence. An updated synthesis is therefore warranted to compare these emerging systems and clarify their clinical and biomechanical relevance.

The aim of this narrative review is to summarize and compare the classification systems for discoid meniscus described in the literature. We focus on morphology-based systems, classifications that additionally incorporate peripheral instability and associated tear characteristics, and imaging-based classifications. For each system, we examine the features included, its intended clinical application, and the reported reproducibility. We also consider the extent to which these classifications reflect structural and biomechanical alterations and may inform tissue preservation, healing potential, and meniscal repair strategies.

## 2. Methods

This narrative review was informed by a literature search conducted in PubMed and Scopus from database inception through June 2026. Search terms included combinations of “meniscus,” “discoid meniscus,” “classification,” “MRI,” “tear,” “biomechanics,” and “treatment.” English-language studies addressing the classification, imaging, biomechanics, or treatment of discoid meniscus were considered, with particular emphasis on pediatric populations. Relevant studies involving adults or biomechanical models were also included when they provided information applicable to discoid meniscus classification or management. The references cited in the identified articles were additionally reviewed. Studies were selected according to their relevance to the objectives of this narrative review; therefore, no formal systematic-review protocol or quantitative synthesis was performed.

## 3. Classifications

Discoid meniscus classification systems fall into four conceptual categories: morphology-only [6,11], morphology-plus-instability [12,13], comprehensive multi-factor systems [14,15], and imaging-based [16]. Only the PRiSM system has undergone formal multicenter reliability testing; Watanabe remains the most widely used, despite failing to capture peripheral instability, which mostly drives symptoms and surgical decision-making.

The Watanabe classification, first described in 1979, categorizes discoid meniscus based on arthroscopic morphology, dividing it into three types (Table 1) [6]. Monllau later expanded this system by introducing a fourth type, describing a ring-shaped meniscus variant with instability and an intact posterior coronal insertion [11]. Although the Watanabe classification provides a useful morphologic framework, it is limited in its clinical applicability. Notably, it does not account for anterior horn instability or the presence of meniscal tears, which are critical factors in symptomatology and surgical decision-making.

### 3.1. Incorporating Instability

**Figure 2:**
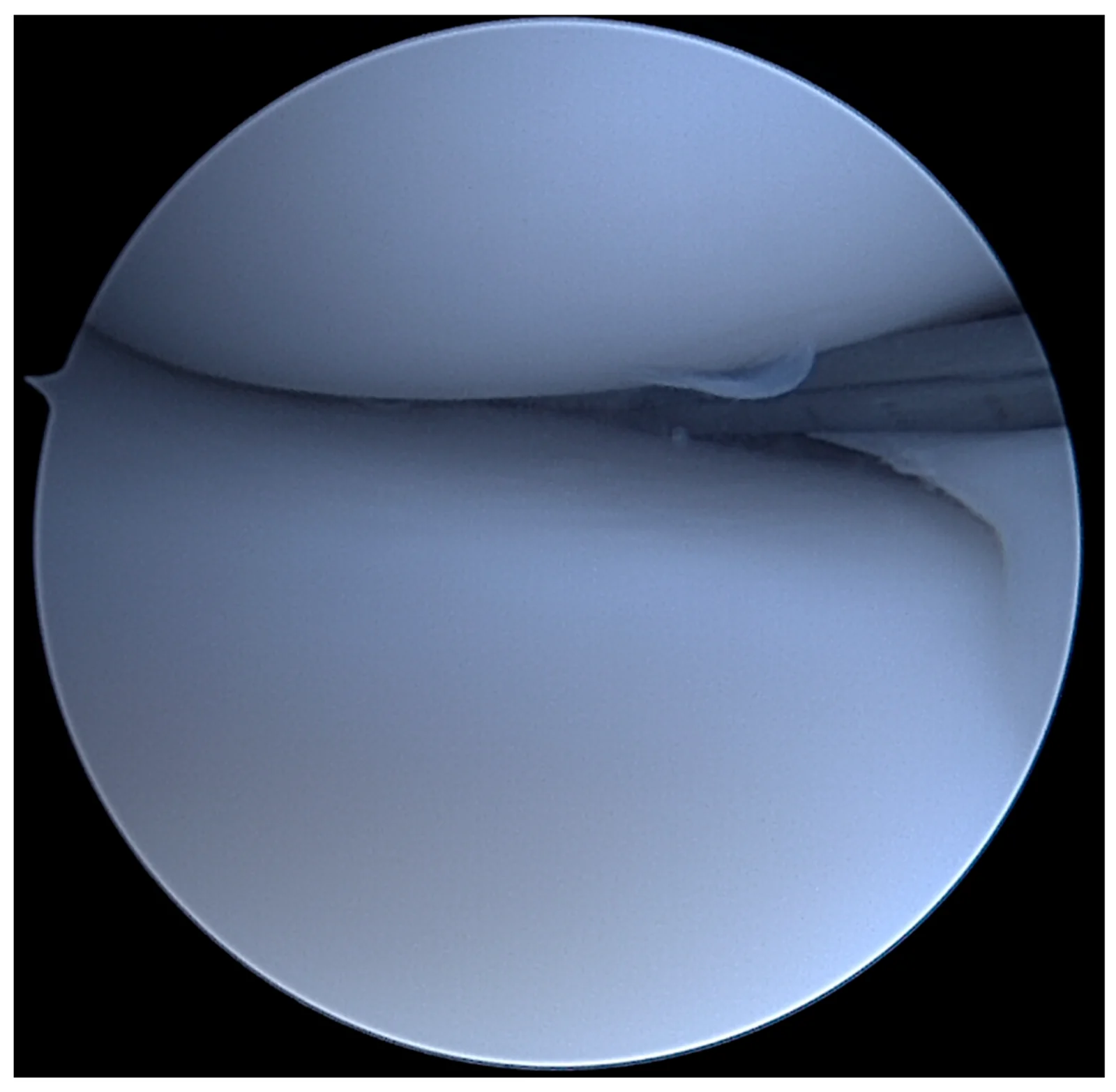
Intraoperative arthroscopic image of a 10-year-old female with a congenital lateral discoid meniscus showing anterior separation from the capsule, with a probe demonstrating instability.

Klingele et al. (2004) highlighted peripheral rim instability as a critical factor that should be incorporated into classification systems for discoid lateral meniscus (Figure 2) [12]. They proposed a modified framework that considers not only meniscal morphology (complete versus incomplete) but also the stability of the peripheral rim. Previous multicenter studies demonstrated that peripheral rim instability was present in 28% to 49% of their DLM cohort at the time of surgery [8,17]. In their study of 128 knees in 112 patients (mean age at surgery, 10.0 years; range, 1 month to 22 years), peripheral rim stability was assessed intraoperatively following saucerization. Stability was evaluated through systematic probing to detect hypermobility or detachment of the remaining meniscal rim, with careful documentation of the location of instability. Peripheral rim instability was significantly more common in complete discoid menisci (38.9%) compared with incomplete discoid menisci (18.2%), suggesting that complete variants are more likely to require meniscal repair. Additionally, unstable discoid menisci were associated with younger age at presentation (8.2 years vs. 10.7 years) [12]. From a biomechanical perspective, peripheral instability disrupts meniscal fixation and compromises the transmission of circumferential hoop stresses, thereby increasing abnormal tissue displacement and local stress concentrations [7]. These findings support the addition of peripheral rim stability to existing classification systems, addressing a key limitation of the Watanabe classification. However, the Klingele classification also has some limitations. Its principal discriminator, peripheral rim instability, requires intraoperative assessment and systematic probing after saucerization. Moreover the system does not characterize tear pattern, lacks formal reliability and prognostic validation, and provides no framework for preoperative imaging-based assessment.

Good et al. (2007) proposed an expanded Watanabe classification that also incorporates meniscus stability [13]. In their study of 30 knees (mean age, 10.1 years), the authors prospectively collected intraoperative data from patients undergoing arthroscopic saucerization with repair as indicated. In addition to classifying discoid menisci according to the Watanabe type, they further characterized each case based on meniscal morphology (complete vs. incomplete), stability (stable vs. unstable), and the location of instability. This study also evaluated several clinically relevant variables, including the presence of preoperative meniscal tears, symptom type and duration, and postoperative outcomes. This expanded framework emphasized the importance of incorporating stability into classification systems, highlighting its role in guiding surgical management and predicting clinical outcomes. By distinguishing stable from unstable tissue, this approach also supports the selection of tissue-preserving procedures aimed at restoring peripheral fixation and meniscal biomechanics. However, the classification was derived from a small Level IV case series, and its instability categories require intraoperative assessment, typically after saucerization. It also omits tear morphology as a graded variable, has not undergone formal reliability testing, and has no validated prognostic value [13].

### 3.2. Comprehensive Multifactorial Systems

Recently, in 2022, Yang et al. proposed a modified classification system designed to serve as a predictive preoperative model to guide surgical planning in patients with discoid lateral meniscus [15]. Building upon the Watanabe classification and prior evidence linking tear patterns to the extent of meniscal resection, the authors evaluated 434 knees and identified 12 distinct combinations based on meniscal stability, morphology, and tear characteristics. In this system, instability is considered the primary determinant of surgical management, with additional emphasis placed on morphologic and tear-related features. Discoid menisci are classified by stability [stable (S0) vs. unstable (S1)], morphology [complete (M0) vs. incomplete (M1)], and tear pattern [no tear (T0), central tear isolated (T1), or central tear with peripheral extension (T2)]. This framework provides a more comprehensive and clinically relevant approach compared to morphology-based systems alone, facilitating improved preoperative assessment and treatment planning. Importantly, the combined evaluation of stability and tear type may help estimate the amount and quality of salvageable tissue, thereby informing the choice between meniscal repair, reshaping, and partial resection. However, the clinical applicability of the Yang et al. (2022) S/M/T classification remains constrained by its derivation from a retrospective, single-center Level III study and the absence of external validation or formal reliability testing [15]. Moreover, it predicts the surgical procedure selected rather than patient outcomes or meniscal survival, while its applicability to preoperative imaging-based assessment remains unestablished [15].

These advances culminated in the development of the PRiSM classification in 2022, created by members of the Pediatric Research in Sports Medicine (PRiSM) society to improve clinical and surgical decision-making for the lateral discoid meniscus (Table 2). The classification was introduced and initially validated within the same multicenter study. This system was developed through a multicenter study analyzing 50 arthroscopic videos of symptomatic discoid menisci collected from 20 academic institutions across North America. The videos were screened for quality, and the sample size was determined to be adequate through an a priori power analysis. The median patient age was 11 years (range, 3–19 years). The PRiSM classification incorporates four key variables: meniscal width, height, stability, and presence of a tear, providing a more comprehensive assessment than prior morphology-based systems. Together, these variables describe not only gross morphology but also features that may affect tissue loading, mechanical competence, and reparability. In the same study, five observers performed the readings for interobserver reliability and three of those five observers performed a second, repeat reading to assess intraobserver reliability. Interobserver agreement was substantial for width, height, stability, tear presence, and tear type (κ 0.61–0.80), but moderate for tear location (κ 0.41–0.60); intraobserver agreement was excellent for peripheral stability (κ 0.81–1.00), substantial for width and height, and moderate for all tear-related variables [14]. These findings support the reproducibility of the system while identifying tear characterization as its least reliable component [14].

The principal limitations of the PRiSM classification are the moderate reliability of some treatment-relevant variables, particularly tear location and, on repeated assessment, tear presence and type. Moreover, validation has been limited to controlled arthroscopic video review rather than live surgery. Although the system may inform procedural selection, its ability to predict patient-reported outcomes or meniscal survival has not been established, and it remains an arthroscopic, primarily descriptive framework [14,18].

### 3.3. Imaging-Based Classifications

Whereas most classification systems for discoid meniscus emphasize arthroscopic findings and clinical presentation, there are emerging imaging-based classification systems [19]. Ahn et al. (2009) proposed a novel preoperative MRI-based classification, which is currently the most widely used [16]. In their study, the authors evaluated preoperative MRIs of 82 knees diagnosed with discoid lateral meniscus (DLM) and categorized findings based on meniscal displacement patterns. The MRI findings were classified into four groups: no shift, anterocentral shift, posterocentral shift, and central shift (Table 3), providing a framework for preoperative assessment that may aid in anticipating intraoperative pathology and guiding surgical planning. The MRI-based shift classification proposed by Ahn et al. (2009) addresses several limitations of the Klingele system by enabling preoperative assessment of displacement patterns that may indicate peripheral detachment or instability [16]. Its categories are also associated with the location of peripheral tears and may help anticipate the need for repair. Rather than replacing the Klingele classification, the Ahn system should be considered a complementary preoperative framework that partially addresses a major limitation of arthroscopy-based classifications: treatment-relevant instability can otherwise be definitively assessed only intraoperatively, often after saucerization [12,16]. Because displacement patterns may reflect abnormal peripheral attachment or instability, this classification can also provide indirect information regarding altered meniscal mechanics and the potential need for repair. However, imaging-based classification systems should be used as an adjunct to arthroscopic findings and clinical presentation, as radiographic features do not always correlate with symptom severity or functional impairment.

MRI relaxation-time measurements have been investigated for the quantitative assessment of meniscal degeneration; however, most evidence derives from nondiscoid menisci and cannot be directly extrapolated to DLM. T2 mapping is currently the only quantitative compositional technique evaluated in a dedicated DLM cohort. Nishino et al. reported longer preoperative T2 relaxation times in symptomatic DLM than in the ipsilateral medial meniscus, higher values on the tear side, and a significant decrease after arthroscopic reshaping [20]. Although these findings suggest that T2 mapping can detect regional and postoperative compositional changes, its ability to predict reparability, healing, or clinical outcomes remains unproven. Conventional MRI therefore remains the reference for DLM diagnosis and preoperative assessment. Future studies should determine whether quantitative MRI and artificial intelligence can provide reliable measures of meniscal structure and tissue quality for inclusion in DLM classification systems and for predicting healing and repair outcomes.

Overall, the evolution of DM classification reflects a transition from simple morphological description toward clinically oriented, multidimensional assessment. The Watanabe and Monllau systems are straightforward and useful for describing anatomical variants but offer limited guidance regarding instability, reparability, or treatment [6,11]. The Klingele and Good classifications improve clinical relevance by incorporating peripheral stability, but they do not include tear pattern, which is another important point in treatment selection. Moreover, their intraoperative nature and lack of external validation limit their application [12,13]. The Yang system further integrates morphology, stability, and tear pattern to support intraoperative planning, but its complexity and external validity require consideration [15]. The PRiSM classification was externally validated, but its prognostic value remains to be established and its four-domain structure makes it difficult to apply widely compared with the Watanabe classification [14]. Conversely, Ahn’s classification facilitates preoperative MRI assessment but should complement rather than replace clinical and arthroscopic evaluation (Table 4) [16].

These differences can be illustrated by considering a complete, thickened DLM with posterior peripheral instability, a longitudinal tear extending to the peripheral rim, and anterocentral displacement on MRI. Under the Watanabe and Monllau systems, this meniscus would be classified as a complete type I variant, without characterization of its instability or tear. The Klingele and Good frameworks would additionally identify the meniscus as unstable and localize the instability posteriorly. In the Yang system, it would be coded as S1-M1-T2, reflecting instability, complete morphology, and peripheral tear extension. The PRiSM classification would describe complete width, abnormal height, posterior instability, and the presence, type, and location of the tear. Finally, the Ahn MRI classification would categorize it according to its anterocentral displacement pattern on preoperative MRI.

Current expert recommendations reflect this complementary relationship: grade discoid menisci by morphology (complete vs. incomplete, from Watanabe/Monllau), by peripheral stability/displacement (Good/Klingele, aided by Ahn’s MRI shift), and by presence of tears (Young)—the combination that the PRiSM system ultimately formalized into a single comprehensive framework.

## 4. Biomechanical Implications

Abnormalities in collagen matrix architecture have long been proposed as a potential explanation for the reduced resistance of discoid meniscal tissue to mechanical loading and its susceptibility to different tear patterns [21,22]. Histological studies have subsequently demonstrated fewer, irregularly oriented, and disorganized collagen fibers in discoid menisci [22,23]. The extent of collagen disruption also appears to be related to meniscal pathology, with greater disorganization observed in torn than in intact discoid lateral menisci and the most severe alterations found in complex tears [7]. These histopathological alterations have important implications for surgical planning, particularly when determining the amount of peripheral tissue to preserve after saucerization and meniscal tear repair. An adequate functional rim is required to maintain the meniscus’s contribution to shock absorption and load distribution. Computational models specific to DLM demonstrate that progressive reduction of residual meniscal width increases tibiofemoral contact stress, with more pronounced deterioration when the preserved rim is reduced below approximately 8–12 mm [24]. These findings provide a biomechanical rationale for limiting tissue resection and preserving or restoring stable peripheral attachments whenever feasible. Tear morphology further influences meniscal mechanics [7]. Finite element studies of nondiscoid medial menisci have demonstrated that tears produce stress concentrations at their margins, with radial tears generating the greatest increase in tissue stress compared with the relatively homogeneous distribution of the intact meniscus [25]. Although these results cannot be directly extrapolated to DLM, they support the inclusion of tear pattern in clinically oriented classification systems. Similarly, finite element models of medial meniscal root repair indicate that repair configuration can affect stress and strain at the fixation site under physiological loading [26]. These findings highlight the potential value of patient-specific computational modeling for comparing repair strategies, although equivalent evidence for DLM remains lacking. Future DLM-specific studies should integrate classification variables with patient-specific finite element models to determine how morphology, tear pattern, instability, residual rim dimensions, and repair configuration influence tissue stress and joint contact mechanics. Prospective studies combining these biomechanical measures with quantitative imaging, tissue assessment, repair healing, and long-term clinical outcomes are needed to determine whether these models provide reliable prognostic information.

## 5. Management

**Figure 3:**
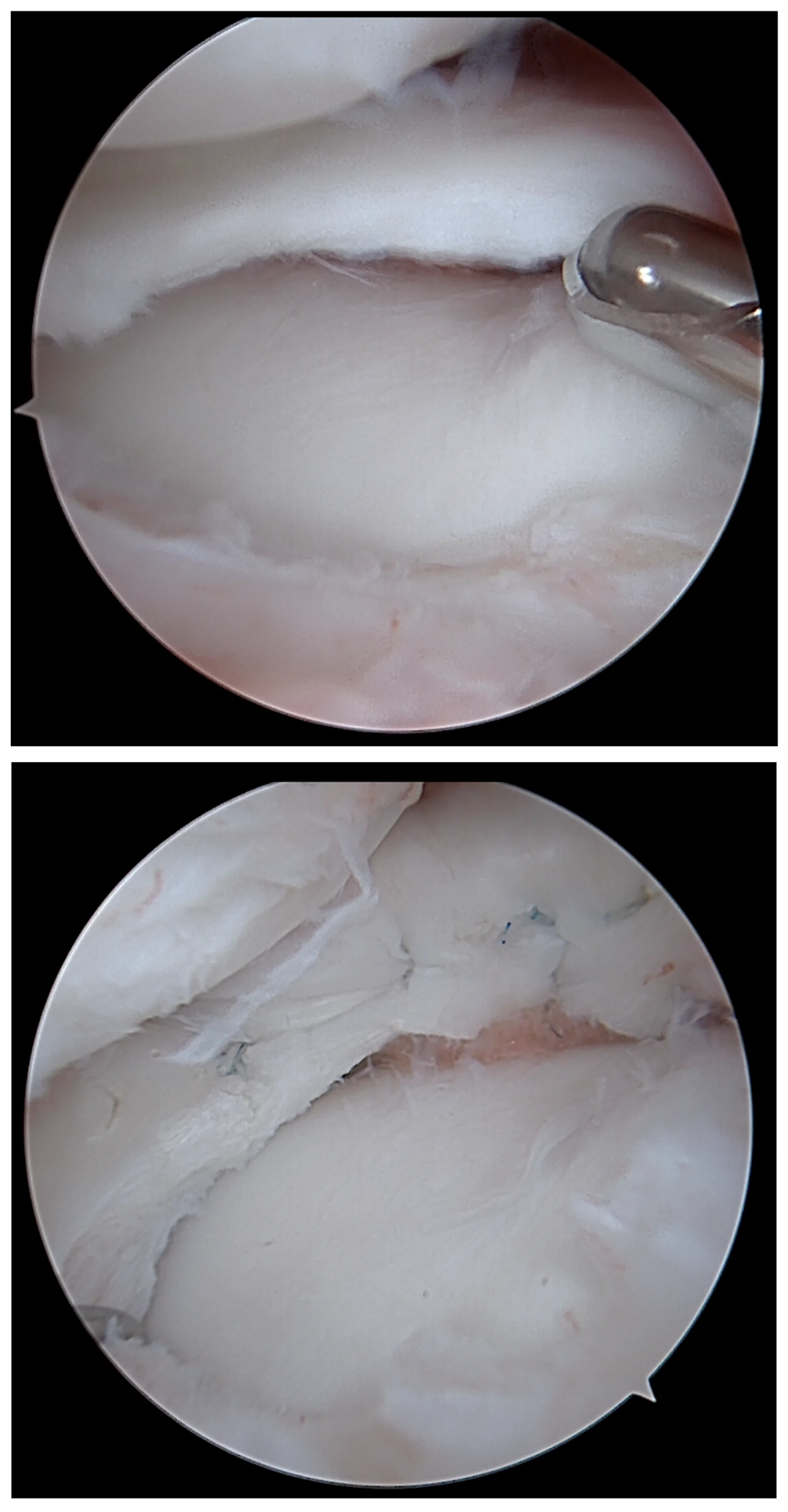
Intraoperative arthroscopic images of a 10-year-old female with a torn congenital lateral discoid meniscus (**top image**) who underwent a central meniscectomy and saucerization with a 5–6 mm circumferential rim remaining (**bottom image**). She also underwent a meniscus repair of the posterior horn using sutures with an all inside approach.

Treatment of a DM depends on symptoms, peripheral stability, tear location, and tissue quality rather than on discoid morphology alone. Nonoperative observation with clinical follow-up is indicated for asymptomatic DM without tears [4]. In symptomatic patients, saucerization is generally performed to remove abnormal central tissue and restore a more physiologic meniscal contour. Preservation of a 6–8 mm peripheral rim is commonly recommended, although some authors advocate preserving up to 10 mm to accommodate postoperative remodeling [9,27,28]. The remaining peripheral rim should then be systematically probed, as instability may become more apparent after reshaping. A stable rim without a residual tear can generally be treated with saucerization alone. In contrast, peripheral rim instability, meniscocapsular disruption, or a repairable longitudinal tear involving the vascular peripheral zone requires saucerization combined with repair (Figure 3). The location of instability—anterior, posterior, or multiregional—guides the site and technique of fixation [18].

The Yang classification links these pathological features to treatment by integrating stability, morphology, and tear pattern. In their study, instability was the strongest determinant of repair: unstable menisci were repaired in 93% of cases, and instability predicted repair with 89% sensitivity and 94% specificity. Tears extending from the central portion to the peripheral rim were also more likely to require repair than isolated central tears [15]. Accordingly, stable S0 menisci with no tear or an isolated central tear (T0–T1) are generally managed with saucerization alone, whereas unstable S1 menisci or those with peripheral tear extension (T2) usually require additional stabilization [15].

The PRiSM classification similarly standardizes intraoperative assessment and supports selection among saucerization alone, saucerization with repair, and additional meniscal debridement or resection. The system characterizes four domains: meniscal width—complete or incomplete; meniscal height—normal or thickened; stability—stable, unstable anteriorly, unstable posteriorly, or unstable both anteriorly and posteriorly; and tear pattern and location. Importantly, width and height are assessed before saucerization, whereas stability and residual tearing are reassessed after reshaping of the peripheral rim. This sequence identifies the tissue that remains mechanically unstable or requires repair [18]. Thus, stability and tear location are the principal treatment-driving variables and have the strongest evidence supporting their predictive value.

Clinically, saucerization combined with repair of peripheral instability has produced favorable intermediate-term outcomes [29]. Nevertheless, recurrent tears and subsequent procedures remain clinically relevant at longer follow-up [30,31], while lateral-compartment degeneration and valgus progression have been reported, particularly after meniscal-sacrificing procedures [30,32]. Nevertheless, across techniques, functional outcomes are favorable, and complication/reoperation rates are low. In a 2026 systematic review (24 studies, 1401 knees), all groups—saucerization alone, saucerization plus repair, and subtotal meniscectomy—showed significant Lysholm improvement; saucerization plus repair had the highest reoperation (8.9%) and complication (6.8%) rates, saucerization alone the lowest (7.3% and 2.7%), differences likely reflecting tear complexity rather than procedural risk [33].

These results support classification systems that incorporate stability, tear characteristics, and the amount and quality of salvageable tissue, while linking each classification category to validated prognostic outcomes.

## 6. Conclusions

In summary, discoid meniscus classification has evolved from systems based primarily on morphology toward more comprehensive frameworks incorporating peripheral instability and associated meniscal tears. Among these systems, PRiSM is the only classification to have undergone formal reliability validation. However, even PRiSM does not associate its classification categories with prognosis, and no current system has been shown to predict healing, reoperation, patient-reported outcomes, osteoarthritis progression, or meniscal survival.

Future classification systems should integrate preoperative imaging features with intraoperative findings. Imaging-based assessment, interpreted in conjunction with the clinical presentation, may help anticipate instability, tear pattern, reparability, and the most appropriate treatment. Arthroscopic evaluation could subsequently confirm the treatment indication and provide additional information with potential prognostic relevance. Machine-learning models could integrate these variables to identify patterns associated with instability, reparability, and treatment outcomes. Patient-specific finite element modeling may estimate load distribution and contact stress, while biological biomarkers of matrix turnover or inflammation could provide complementary information on tissue degeneration and healing potential.

## Figures and Tables

**Table 1:** Summary of Watanabe classification.

Type	Classification	Description	Stability	Posterior Coronal Insertion
I	Complete Discoid	Covers entire tibial plateau	Mechanically stable	Intact
II	Incomplete Discoid	Covers up to 80% of the tibial plateau	Stable on palpation	Intact
III	Wrisberg Variant	Normal or slightly discoid shape	Unstable	Absence

**Table 2:** PRiSM discoid meniscus classification.

**Meniscal Width**	
W0—Normal	
W1—Incomplete	Wider than normal, <90% of tibial plateau covered,
W2—Near-Complete/Complete	≥90% of tibial plateau covered
**Meniscal Height**	
H0—Normal	Thickness appears identical to that of a normal meniscus
H1—Abnormal	Thickness is greater than that of a normal meniscus
**Stability**	
S0—Normal	
SA—Abnormal * (Anterior)	
SP—Abnormal * (Posterior)	
SAP—Abnormal * (Anterior + Posterior)	
**Non-Vertical Tears** **(Tear Type/Location)** **	
T0—No Tear, or Tear in the central portion/saucerization zone	
THA—Horizontal Tear *** (Anterior)	
THP—Horizontal Tear *** (Posterior)	
THAP—Horizontal Tear *** (Anterior + Posterior)	
TDA—Degenerative/Complex/Radial Tear (Anterior half)	
TDP—Degenerative/Complex/Radial Tear (Posterior half)	
TDAP—Degenerative/Complex/Radial Tear (Anterior + Posterior half)	

PRiSM = Pediatric Research in Sports Medicine Society. * Abnormal stability due to absent, lax/deficient meniscocapsular attachments (meniscal translation beyond the apex of the lateral femoral condyle), or peripheral vertical tears of the meniscus/meniscocapsular junction. ** Tear location classified as anterior or posterior by predominant involvement; combined if extending across the midbody. *** Horizontal cleavage defined as >5 mm width and >50% depth or extension into the red-red zone. Bold text denotes the four classification domains (meniscal width, height, stability, and tear type/location); values listed beneath each are the corresponding classification codes.

**Table 3:** Summary of Ahn et al. MRI-based discoid meniscus classification.

Class	Peripheral Rim of DLM	Direction of Meniscus Displacement
No shift	Peripheral portion of DLM is attached to the joint capsule	None
Anterocentral Shift	Peripheral portion of posterior horn is detached from capsule	Anteriorly or Anterocentrally
Posterocentral Shift	Peripheral portion of anterior horn detached from the capsule	Posteriorly or Posterocentrally
Central Shift	Peripheral portion of posterolateral DLM torn or lost	Centrally (toward intercondylar notch)

**Table 4:** Summary of most commonly used discoid meniscus classifications.

Classification	Basis/Categories	Diagnostic Utility	Reproducibility	Ease of Clinical Application	Prognostic Value	Key Limitations
Watanabe (1979) [6]	Arthroscopic/gross morphology: complete (I), incomplete (II), Wrisberg (III, lacking posterior tibial attachment)	Foundational, universally understood; defines shape and the hypermobile Wrisberg variant	Not formally validated by kappa; long clinical familiarity	High—three category scheme	Limited—does not systematically capture instability or tear pattern that drive symptoms/outcomes	Omits peripheral rim instability, tear pattern, and height; Wrisberg type rarely encountered in some series
Monllau (1998) [11]	Adds a 4th ring-shaped variant to Watanabe	Recognizes a rare morphological variant missed by Watanabe	Not validated; based on isolated reports	High conceptually—rarely applied	Minimal established prognostic data	Describes an uncommon incidental variant; no instability or tear data
Klingele (2004/2003) [12]	Watanabe morphology + peripheral rim instability by location (anterior/middle/posterior thirds)	Highlighted high prevalence of rim instability (28%), reframing surgical assessment	Not formally kappa-tested	Moderate—arthroscopic probing required	Instability more common in complete types and younger patients; guides repair vs. saucerization	Instability assessed intraoperatively only; no imaging correlate; no tear-type stratification
Good (2007) [13]	Complete/incomplete + subclassified by presence and location of capsular instability (anterior-horn predominant)	Emphasized high anterior-horn instability (53%), informing repair technique choice	Small single series (Level IV); not reliability-tested	Moderate—arthroscopic probing required	Guides repair approach (outside-in anterior vs. inside-out posterior); short-term results good	Small cohort, short follow-up; no validated reproducibility
Ahn (2009) [16]	Preoperative MRI by peripheral shift: no shift, anterocentral, posterocentral, central shift	Adds preoperative planning value; shift types predict peripheral tears and need for repair	Reported inter-/intraobserver correlation coefficients; standard MRI has ~40–50% false-negative rate for instability	Moderate—requires MRI expertise; final decision still arthroscopic	Shift-type predicts peripheral tears/repair (55% vs. 28%); correlates with Wrisberg ligament anatomy	Standard MRI misses dynamic instability (improved by dynamic protocols); confirmation still needs arthroscopy
Yang (2022) [15]	Three-axis arthroscopic: stability (S0 stable/S1 unstable), morphology (M0 complete/M1 incomplete), tear (T0 none/T1 non-peripheral/T2 peripheral)	Directly predicts need for repair; instability alone 89% sensitive/94% specific for repair decision	Designed for surgical decision-making; formal interobserver kappa not reported in retrieved data	High—three intuitive binary/ternary axes applied after saucerization	Strong: unstable menisci 114× higher odds of repair; peripheral tears 6.4× higher odds	Reproducibility not formally kappa-tested; single-center derivation; outcome-linked validation still maturing
PRiSM group (2022, Lee et al.) [14]	Comprehensive arthroscopic: meniscal width, height, peripheral stability, and tear (presence/type/location) via stepwise dual-portal exam	Most complete description of the pathomorphologic spectrum; designed to overcome prior systems’ gaps	Only system with formal multicenter testing: interobserver substantial for width/height/stability/tear presence/type, moderate for tear location; intraobserver excellent for stability	Moderate—requires standardized stepwise exam and 4-factor assessment	Directly informs saucerization vs. repair; prognostic outcome data still maturing	Newer, limited longitudinal outcome data; more complex than Watanabe

## Data Availability

No new data were created or analyzed in this review; all data discussed are available within the cited references.

## References

[1] Masouros S.D., McDermott I.D., Amis A.A., Bull A.M.J. (2008). Biomechanics of the Meniscus-meniscal Ligament Construct of the Knee. Knee Surg. Sports Traumatol. Arthrosc..

[2] Kim J.H., Ahn J.H., Kim J.H., Wang J.H. (2020). Discoid lateral meniscus: Importance, diagnosis, and treatment. J. Exp. Orthop..

[3] Logan C.A., Tepold F.A., Kocher S.D., Feroe A.G., Micheli L.J., Kocher M.S. (2021). Symptomatic discoid meniscus in children and adolescents: A review of 470 cases. J. Pediatr. Orthop..

[4] Tapasvi S., Shekhar A., Eriksson K. (2021). Discoid lateral meniscus: Current concepts. J. ISAKOS.

[5] Kelly B.T., Green D.W. (2002). Discoid Lateral Meniscus in Children. Curr. Opin. Pediatr..

[6] Watanabe M., Takeda S., Ikeuchi H. (1979). Atlas of Arthroscopy.

[7] Choi Y.H., Seo Y.J., Ha J.M., Jung K.H., Kim J., Song S.Y. (2017). Collagenous ultrastructure of the discoid meniscus: A transmission electron microscopy study. Am. J. Sports Med..

[8] Sheasley J.A., Kirby J.C., Niu E.L., Gopalan M., Carsen S., Stinson Z.S., Finlayson C.J., Nault M.-L., Lee R.J., Haus B.M. (2024). Characteristics and outcomes of operatively treated discoid lateral meniscus in pediatric and young adult patients: A multicenter study. Am. J. Sports Med..

[9] Turati M., Crippa M., Waldner J., Bigoni M., Green D.W., Accadbled F. (2025). Surgical management of the discoid meniscus. Knee.

[10] Amiri S., Mirahmadi A., Parvandi A., Moshfegh M.Z., Abatari S.P.H., Farrokhi M., Hoseini S.M., Kazemi S.M., Momenzadeh K., Wu J.S. (2024). Excellent accuracy of magnetic resonance imaging for diagnosis of discoid meniscus tears: A systematic review and meta-analysis. J. Exp. Orthop..

[11] Monllau J.C., Leon A., Cugat R., Ballester J. (1998). Ring-shaped lateral meniscus. Arthroscopy.

[12] Klingele K.E., Kocher M.S., Hresko M.T., Gerbino P., Micheli L.J. (2004). Discoid lateral meniscus: Prevalence of peripheral rim instability. J. Pediatr. Orthop..

[13] Good C.R., Green D.W., Griffith M.H., Valen A.W., Widmann R.F., Rodeo S.A. (2007). Arthroscopic treatment of symptomatic discoid meniscus in children: Classification, technique, and results. Arthroscopy.

[14] Lee R.J., Nepple J.J., Schmale G.A., Niu E.L., Beck J.J., Milewski M.D., Finlayson C.J., Joughin V.E., Stinson Z.S., Pace J.L. (2022). Reliability of a new arthroscopic discoid lateral meniscus classification system: A multicenter video analysis. Am. J. Sports Med..

[15] Yang B., Kocher M.S., Fabricant P.D., Kocher S.D.B., Williams K.A., Kocher M.S. (2022). Utility of stability and tear location in a classification system for discoid meniscus surgical planning. J. Pediatr. Orthop..

[16] Ahn J.H., Kim K.I., Wang J.H., Jeon J.W., Cho Y.C., Lee S.H. (2009). A novel magnetic resonance imaging classification of discoid lateral meniscus based on peripheral attachment. Am. J. Sports Med..

[17] Silverstein R.S., McKay S.D., Coello P., Pupa L., Latz K., Kemper W.C., Adsit E., Wilson P.L., Albright J., Algan S. (2023). Relationship between age and pathology with treatment of pediatric and adolescent discoid lateral meniscus: A report from the SCORE multicenter database. Am. J. Sports Med..

[18] Beck J.J., Pandya N.K., Allahabadi S. (2025). Discoid lateral meniscus evaluation and treatment. Arthroscopy.

[19] Samoto N., Kozuma M., Tokuhisa T., Kobayashi K. (2002). Diagnosis of discoid lateral meniscus of the knee on MR imaging. Magn. Reson. Imaging.

[20] Nishino K., Hashimoto Y., Nishida Y., Yamasaki S., Nakamura H. (2023). Arthroscopic surgery for symptomatic discoid lateral meniscus improves meniscal status assessed by magnetic resonance imaging T2 mapping. Arch. Orthop. Trauma Surg..

[21] Setton L.A., Guilak F., Hsu E.W., Vail T.P. (1999). Biomechanical factors in tissue engineered meniscal repair. Clin. Orthop. Relat. Res..

[22] Papadopoulos A., Kirkos J.M., Kapetanos G.A. (2009). Histomorphologic study of discoid meniscus. Arthroscopy.

[23] Atay O.A., Pekmezci M., Doral M.N. (2007). Discoid meniscus: An ultrastructural study with transmission electron microscopy. Am. J. Sports Med..

[24] Liu W., Sun X., Liu W., Liu H., Zhai H., Zhang D., Tian F. (2022). Finite element study of a partial meniscectomy of a complete discoid lateral meniscus in adults. Med. Eng. Phys..

[25] Kurt C., Gok A. (2026). Effect of knee joint meniscus tears on joint cartilage contact and pressure with finite element analysis. Biomedicines.

[26] Kurt C., Ozsoy M.H., Gok A., Inal S., Gok K. (2025). Alternative surgical technique for the repair of meniscus root tears using finite element analysis. Int. J. Numer. Method BioMed Eng..

[27] Nishino K., Hashimoto Y., Nishida Y., Nakamura H. (2021). Morphological changes in the residual meniscus after reshaping surgery for a discoid lateral meniscus. Am. J. Sports Med..

[28] Heitz P.H., Pauyo T., Beck J.J., Niu E.L., Lee R.J., Pace J.L., Schmale G.A., Carsen S., Heyworth B.E., Milewski M. (2025). Variation in arthroscopic treatment of discoid lateral meniscus and postoperative restrictions in children: Results of a multicenter meniscus study group survey. Orthop. J. Sports Med..

[29] Perkins C.A., Busch M.T., Christino M.A., Willimon S.C. (2021). Saucerization and repair of discoid lateral menisci with peripheral rim instability: Intermediate-term outcomes in children and adolescents. J. Pediatr. Orthop..

[30] Sabbag O.D., Hevesi M., Sanders T.L., Camp C.L., Dahm D.L., Levy B.A., Stuart M.J., Krych A.J. (2019). High rate of recurrent meniscal tear and lateral compartment osteoarthritis in patients treated for symptomatic lateral discoid meniscus: A population-based study. Orthop. J. Sports Med..

[31] Lins L.A.B., Feroe A.G., Yang B., Williams K.A., Kocher S.D., Sankarankutty S., Micheli L.J., Kocher M.S. (2021). Long-term minimum 15-year follow-up after lateral discoid meniscus rim preservation surgery in children and adolescents. J. Pediatr. Orthop..

[32] Cho J.H., Nam H.S., Park S.Y., Ho J.P.Y., Lee Y.S. (2024). Arthroscopic meniscal repair and meniscectomy for adult discoid lateral meniscus results in progression to valgus alignment and lateral compartment degeneration compared with nonoperative treatment and nondiscoid lateral meniscus. Arthroscopy.

[33] Hartman H., Lessiohadi N., Saharan S., Saraf S.M., Christiansen M., Mulcahey M.K. (2026). Outcomes of saucerization with or without repair for symptomatic discoid lateral meniscus in pediatric patients: A systematic review. Arthroscopy.

